# 

**Published:** 1883-06-20

**Authors:** 


Entered at the Post-Office at Atlanta, as second-class matter.

VOL. XIII. JUNE 20, 1883. NO. 6.

### THE SOUTHERN MEDICAL RECORD:

### A MONTHLY JOURNAL OF PRACTICAL MEDICINE.

**Terms: $2.00 per Annum, in Advance; 4 Copies $6.00; Single Copies 20 Cents.**

*"QUICQUID PRÆCIPIES ESTO BREVIS."*

